# Attitude, Opinions, and Working Preferences Survey among Pet Practitioners Relating to Antimicrobials in India

**DOI:** 10.3390/antibiotics11101289

**Published:** 2022-09-22

**Authors:** Kushal Grakh, Dinesh Mittal, Tarun Kumar, Swati Thakur, Diksha Panwar, Lokender Singh, Manesh Kumar, Naresh Jindal

**Affiliations:** 1 Department of Veterinary Public Health and Epidemiology, Lala Lajpat Rai University of Veterinary and Animal Sciences, Hisar 125004, India; 2 Department of Veterinary Clinical Complex (VCC), Lala Lajpat Rai University of Veterinary and Animal Sciences, Hisar 125004, India; 3 Department of Veterinary Physiology and Biochemistry, Lala Lajpat Rai University of Veterinary and Animal Sciences, Hisar 125004, India; 4 Department of Animal Biotechnology, Lala Lajpat Rai University of Veterinary and Animal Sciences, Hisar 125004, India

**Keywords:** antimicrobial resistance, India, survey, pet practitioner, companion animals

## Abstract

The indiscriminate usage and overuse of antimicrobials in pets or companion animals are underlying causes of antimicrobial resistance (AMR). Despite the multi-faceted global challenge presented by antimicrobial resistance, very few studies have appraised pet practitioners’ factors, such as written policy on antimicrobials, dose rate prescribed, use of critically important antimicrobials, and antimicrobial prescription in clean surgical procedures, which can contribute to AMR. In the present study, an online cross-sectional survey among randomly selected pet practitioners (*n* = 104) of various Indian provinces and union territories was conducted using a questionnaire comprising 33 closed-ended questions on different parameters, viz., the dosage regimen and level of compliance towards guidelines of the World Health Organization (WHO), other relevant veterinary associations, and their opinion while prescribing antimicrobials. Almost every practitioner of the 104 respondents had revealed the difficulties with owner compliance; i.e., incomplete course of the antibiotics, inappropriate follow-ups, and improper care of the sick animals. The majority of practitioners (95%) reported self-prescription of antimicrobials by the owner before presenting the pet(s) to the veterinary clinic, whereas more than half of the respondents (64%) revealed unavailability of antibiogram facilities. Furthermore, a large number (76%) of practitioners stated empirical treatment based on their experience as the main criteria for antimicrobial choice in the absence of timely results from the laboratory. Although non-necessitated use of antimicrobials in clean surgical procedures has been claimed, surprisingly, the majority of pet practitioners (97%) reported their use to reduce the post-operative complications. The use of the highest priority, critically important antimicrobials (HPCIA) listed by the WHO for humans, particularly quinolones and third-generation cephalosporin, also has been reported for different infections. The treatment durations were nearly as per the recommended guidelines issued by the Danish Small Animal Veterinary Association (DSAVA) for different ailments. Analysis using chi-square tests exhibited a significant correlation between less experienced veterinarians (less than 5 years) and prescription of antimicrobials restricted for critically important infections in human medicine. However, there seems to be no association between the experience of the practitioner and the further studied parameters, namely, antimicrobial regimen prescription, weighing the animals before prescription, dose rate calculation, and antimicrobial selection and use after clean surgical operations. The findings suggest periodic awareness campaigns among practitioners regarding the implementation of the official guidelines, the need for systematic surveillance of AMR, awareness among pet owners about antimicrobial resistance, and the importance of rational use of antimicrobials on their pets.

## 1. Introduction

Antimicrobial resistance (AMR) has become an increasingly important global concern, arising due to the irrational use of antimicrobials in both human and veterinary medicine. Of late, the use of antimicrobials for the treatment of infectious diseases and/or other ailments in companion animals may be necessary; however, emergence of bacterial populations resistant to several classes of antimicrobials is persistently increasing [1,2]. Besides creating a hindrance in prescribing effective antimicrobial treatment to the patient, AMR can be transmitted to other animals and humans by virtue of horizontal genetic material transfer, further complicating the treatment of other diseases [3]. Moreover, the close contact of pets with their owners and surrounding environment (kitchen, floor, beds) provides the opportunity for the transmission of multidrug-resistant organisms between humans and their pets [4,5]. The bacterial pathogens calling for antimicrobial usage in companion animals, particularly canines, are *Staphylococcus* spp., *Salmonella* spp., *Escherichia coli*, *Klebsiella pneumoniae*, and *Campylobacter* spp. [6,7]. A recent study reported that more than 83% of broad-spectrum antimicrobials and around 71% of critically important antimicrobials for human medicine are used for the treatment of dogs and cats in European countries [8]. Antimicrobial usage in pet animals in India is not ascertained but is expected to be lesser in comparison to livestock [9]. The inappropriate use of antimicrobials in human as well as animal populations is a major issue, mainly due to unregulated quackery practices in the country [10]. With a huge human population and millions of wandering stray animals, including dogs, people are surrounded by a high microbial burden, viz., massive amounts of fecal matter and manure in their environment [11]. The AMR issue is equally important and prevalent in animals, but never emphasized to the extent as in humans. India is among the largest consumers of antimicrobials for livestock in the world. [12]. The information on the practice of antimicrobials and current status of antimicrobial susceptibility for bacterial pathogens at local levels will provide insight into the probable drivers of antimicrobial resistance (AMR) in the pet industry, which will ultimately aid in formulating strategies for its containment. Despite the multi-faceted global challenge presented by antimicrobial resistance, studies pertaining to the rationale behind the prescription of antimicrobials by pet practitioners are lacking, especially in India.

In view of the above, the present study was envisaged with the primary aim to collect first insights into the rationale for antimicrobials prescription, including the use of ailment-specific antimicrobials and treatment duration, followed by pet practitioners in India. Additionally, it was hypothesized that antimicrobial usage practices among pet practitioners in India are similar to those of the Danish Small Animal Veterinary Association (DSAVA), whose data were used for analysis and comparison in the present study [13]. The collected information may help in better understanding the current prescribing patterns and act as the first step in providing guidance for and targeting of antimicrobial stewardship (AMS) interventions. As per the literature appraisal, and to the best of our knowledge, this is the first survey of its kind from India.

## 2. Results

A total of 104 (52.0%) pet practitioners responded to the questionnaire. The respondent practitioners were from 13 different states and two Union Territories of India.

### 2.1. Demographic Information

**Figure 1:**
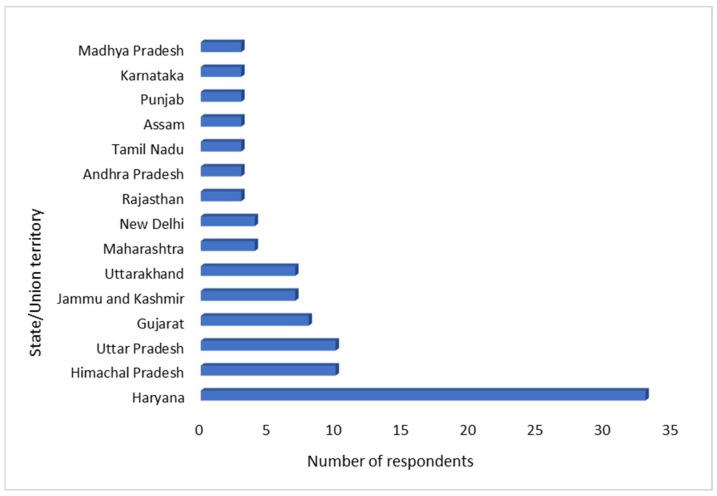
Distribution of respondents according to the states/union territories of India.

Most of the respondents were from the state of Haryana (*n* = 33, 31.7%) followed by Himachal Pradesh, Uttar Pradesh (*n* = 10, 9.6%), and others, as depicted in Figure 1. The state of Haryana has 22 districts with nearly the same number of major urban cities. All the respondents in the current study had clinics in urban cities. The majority of the respondents were male (*n* = 74, 71.1%), and the mean age of the respondents was 31.34 ± 8.6 years. The majority of the respondents (*n* = 80, 76.92%) were 24–34 years old followed by the 35–50 years old age group (*n* = 19, 18.3%) and more than 50 years old age group (*n* = 5, 4.8%). The maximum number of respondents (*n* = 56, 53.8%) were the practitioners engaged in clinical practice at their own clinic/hospital. A total of 71 (68.2%) respondents were had less than or equal to 5 years of professional experience in small animal practice. Responses received from all the categories are provided in Table 1, Table 2 and Table 3.

### 2.2. Antimicrobial Usage

More than half of the pet practitioners (*n* = 58, 56%) reported no written policy or standard operating procedure with respect to antimicrobial use. The majority of the veterinarians (64%) were lacking a facility for antimicrobial sensitivity testing at their place of practice/clinic. A total of 61% of pet practitioners responded that they followed the recommended dosage rate for antimicrobials as per the ailment rather than any other perception. Most veterinarians (*n* = 99, 95%) revealed that pet owners (65% “sometimes”, 30% “frequently”) present the case to clinic after starting systemic antibiotics medication to their pets at their own level. The majority of the (67%) veterinarians responded that they rarely prescribe a combination of different antimicrobials, whereas around 29% reported frequent use of such combinations in their practice. Every veterinarian reported owner compliance challenges (6% “always”, 37% “frequently” and 56% “sometimes”) in terms of completion of antimicrobial treatment course, except one. Concerning the source of choosing an antimicrobial for a specific condition, most veterinarians (*n* = 44, 42%) answered that their own professional experience is their method of choice followed by bibliographic sources, including veterinary indexes/subject books (*n* = 41, 39%). The majority of the veterinarians (*n* = 97, 94%) admitted that the antimicrobials were administered whilst antimicrobial sensitivity test results were awaited (53% “sometimes”, 32% “frequently” and 9% “always”) and a few (*n* = 7, 6%) responded that this practice was never followed. A total of 72 (69%) respondents answered that they use antimicrobials exclusively meant for human use (52% “sometimes”, 12% “frequently” and 5% “always”) in their practice, while 32 (31%) respondents reported that they never use such antimicrobials in their practice. The majority of the veterinarians (*n* = 101, 97%) declared that they use antimicrobials in clean surgical procedures. Out of these, 77 practitioners responded use of antimicrobials in more than 50% of such cases presented to them. Most veterinarians stated that frequent postoperative complications are the reason behind usage of antimicrobials in clean surgical operations. Nearly half of the veterinarians (*n* = 54, 52%) reported that selection of the antimicrobials is done solely on the basis of experience and susceptibility testing is performed only in non-responsive cases.

An analysis of the collected data indicated that for skin infections and sepsis, penicillin was mainly used, whereas, for ear- and/or eye-related infections and urinary tract infections, aminoglycosides and quinolones were the commonly used antimicrobials, respectively. The sulphonamides were mainly prescribed for gastrointestinal tract (GIT) infections. The survey further indicated that for reproductive tract-related ailments, 3rd- and 4th-generation cephalosporins were mainly prescribed, and quinolones were the drug of choice for respiratory tract infections (Table 2). Macrolides and carbapenems, including meropenem and imipenem, were also used by some of the practitioners, mainly for respiratory and sepsis-related conditions.

**Table 2:** Frequency of antimicrobials used by practitioners for different organ systems.

	Skin	Ear	Urinary	GIT	Reproductive	Respiratory	Sepsis	Eye
Penicillins	**20**	17	20	26	28	23	**34**	16
Cephalosporins 1st and 2nd generations	17	18	20	19	20	23	22	17
Cephalosporin 3rd and 4th generations	19	13	25	28	**33**	28	33	13
Aminoglycosides	11	**19**	19	20	23	21	26	**18**
Quinolones	14	14	**27**	19	31	**30**	25	**18**
Tetracyclines	9	8	7	17	20	17	19	13
Sulphonamides	8	6	12	**30**	7	7	10	6
Lincosamides	13	3	4	4	5	7	7	4
Macrolides	5	3	6	7	9	11	8	8
Imipenem/Meropenem	4	2	3	5	7	5	11	3
Nitroimidazole	2	1	7	14	19	2	9	2
EVP ^#^	2	1	2	1	1	1	-	1

The cells in bold represent the preferred antimicrobials for each organ system. ^#^ Ethnoveterinary products.

### 2.3. Duration of Treatment

Based on the specific organ system involved, pet practitioners reported that different treatment durations were required for completion of the treatment regimen (Table 3). The treatment duration was highly variable for skin and ear infections. For skin infections, most practitioners (60%) reported treatment for more than 7 days and around 37% reported treatment for 3–7 days. For ear infections, 49% of practitioners reported the treatment duration as 3–7 days and around 43% reported it being more than 7 days. Most of the practitioners (75%, 71%, and 58%) were of the opinion that animals treated against GIT, respiratory, and reproductive infections, respectively, required a treatment regimen of 3–7 days. More than half of the practitioners (54%, 55%, and 56%) reported 3–7 days treatment duration for urinary, sepsis, and eye ailments, respectively.

**Table 3:** Frequency and percentage of responses of the duration of treatment (columns) for different organ systems (rows). The values parentheses are the percentages.

Organ System	Treatment Length				
	Less than 3 Days	3–7 Days	8–14 Days	15–21 Days	Over 21 Days
Skin	3 (2.9)	**39 (37.5)**	26 (25.0)	23 (22.1)	13 (12.5)
Ear	8 (7.7)	**51 (49.0)**	32 (30.8)	9 (8.6)	4 (3.8)
GIT	13 (12.5)	**78 (75.0)**	9 (8.6)	4 (3.8)	
Reproductive	4 (3.8)	**60 (57.7)**	27 (25.9)	12 (11.5)	1 (0.9)
Respiratory	4 (3.8)	**74 (71.1)**	17 (16.3)	7 (6.7)	2 (1.9)
Urinary	2 (1.9)	**56 (53.8)**	27 (25.9)	15 (14.4)	4 (3.8)
Sepsis	4 (3.8)	**57 (54.8)**	33 (31.7)	9 (8.6)	1 (0.9)
Eye	15 (14.4)	**58 (55.8)**	19 (18.3)	7 (6.7)	5 (4.8)

The cells in bold represent the most used duration of treatment for every organ system.

### 2.4. Correlation of Different Variables with Years of Experience

From the statistical analysis using chi-square tests, it was observed that fewer years of experience (less than five) was significantly associated with a higher prescription of human antimicrobials to pet animals. However, there was no significant correlation between the experience years of a practitioner (less than 5 years, 5–15 years, and >15 years) and the written antimicrobial policy at clinics, weighing the animals before prescription, method of selecting antimicrobials, dose rate application, antimicrobials use after clean surgical operations, and other factors as listed (Table 4).

## 3. Discussion

The study seems to be the first of its kind exploring the pattern of antibiotic usage in the treatment of ailing pets in Indian settings. An encouraging number of veterinarians (*n* = 104, 52.0%) responded to the questionnaire survey with maximum responses from the state of Haryana. The exact or approximate representation of canine/feline veterinarians used in the study cannot be ascertained as there are no legitimate data available on the number of small animal clinics/hospitals in private sectors. The sample size used in this study is significant as compared to similar studies conducted in other countries [2,14]. However, further comprehensive studies with more participants across different states of India are warranted for more detailed insight into the problem.

In the present study, around 44% of veterinarians admitted that they have a written antimicrobial policy for their practice, which is higher compared to South Africa and Denmark, where the policy was under practice among 27% and 30% of practitioners, respectively [13,15]. Surprisingly, around 97% of practicing veterinarians in the UK are prescribing antimicrobials without any prior written protocols [16]. A well-written antimicrobial prescription protocol might serve as an important tool to ensure rational treatment, as well as in limiting the prescription of antimicrobials of critical clinical importance to human medicine. A large number of Indian veterinarians are using written protocols even in the absence of guidelines on this aspect by any veterinary organization. Due to the lack of any specified guidelines, practitioners usually develop their own practices/protocols, which may result in variability in terms of antimicrobial prescription and use of last-resort or critically important antimicrobials reserved for humans. Additionally, a large number of practitioners (40%) reported that they calculate the dose rate of antimicrobials as per general principles or leaflets and not as per the condition of the animal(s), which might possibly lead to overdosing or underdosing of antimicrobials. The major concern found in this study was self-administration of next-generation antimicrobials by the majority of pet owners before the pet is presented to a veterinarian. It appears that pet owners might be using leftover antimicrobials available at home (either human or veterinary medicine) without the knowledge of antimicrobial spectrum or resistance profile. Occasionally, antimicrobials in India are available over-the-counter owing to the lack of strict legislation and regulation on antimicrobial sales [11]. The use of different combinations of antimicrobials is not a frequent practice in India, similar to Italy [17] but differing from the UK, the latter reporting frequent prescription of such combinations [11]. Antimicrobial combinations can help to achieve synergistic or additive results, thereby preventing the emergence of AMR [18]. Almost every veterinarian reported owner-compliance challenges, which might be due to a lack of awareness among pet owners prompting them to stop the treatment once clinical sign abates [13]. This aspect needs further investigation by involving pet owners, as it will help to decide the need to educate pet owners accordingly.

Only 7% of the respondents answered that they never prescribe antimicrobials before the results of antimicrobial susceptibility testing (AST), whereas 93% responded that they need to prescribe the antimicrobials while waiting for the AST results with variable frequency (from “sometimes” to “always”). It indicates that the first choice of antimicrobials is usually made empirically, as most of the respondents answered that their antimicrobial selection is based on their professional experience followed by a bibliography (veterinary index/subject books). The use of antimicrobials solely on the basis of experience need to be avoided unless there is a life-threatening infection demanding immediate antimicrobial use [19]. The majority of the veterinarians in this study reported a lack of AST facilities at their clinics, which limits the rational prescription of antimicrobials and promotes the empirical treatment. Moreover, the pet owners from semi-urban or rural areas of India expect low-cost treatment for their pets, forcing veterinarians to avoid microbiological analysis and AST (personal communication). For cases where empirical use of antimicrobials is unavoidable, the pet practitioners must have up-to-date information on local resistance profiles to narrow down the choice of antimicrobial(s) [14]. Alternatively, adopting novel technologies, such as matrix-assisted laser desorption/ionization time-of-flight mass spectrometry (MALDI-TOF-MS), and automated instruments, such as the BD Phoenix Automated Microbiology System (BD Diagnostics, Billerica, MA, USA), Vitek 2 System (bioMérieux, Marcy-l-Etoile, France), can not only reduce the time required for estimation of AST but will also help to process large batches of samples, simultaneously [20,21]. However, the high cost of these equipment and expectation of the owner in terms of low-cost treatments seems to be a great hindrance on the way. Most of the veterinarians (90%) reported that pets returning to the clinics were due to antimicrobial treatment failure (76% “sometimes” and 14% “frequently”). This might explain the need for maintaining the clients’ records with respect to antimicrobials prescription. A higher number of practitioners (87%) in the current study were not maintaining such records, of which 13% did not consider it necessary, citing that it consumes more time, cost, and labor. The majority of the respondents weigh the animals before prescribing antimicrobials, but the frequency of weighing varied from practitioner to practitioner. The weighing of the animal before an antimicrobial prescription is a good clinical practice, as it would allow an accurate dose rate administration to the patient [22].

The responses received on the types of antimicrobials used and the duration of treatment for specific organ systems were of serious concern. There are no guidelines to compare the findings of the present survey in India. So, the guidelines formulated by the Companion Animal Group, Danish Veterinary Association [13], were used to compare to the responses provided by the veterinarians. In the survey, practitioners reported that penicillin and cephalosporins of the 1st, 2nd, 3rd, and 4th generation are the preferred choices for the management of skin infections. These responses are encouraging as they are in line with the guidelines formulated for such infections. Cutaneous infections present a serious threat to animal health due to the increased risk of treatment failure, mostly due to methicillin-resistant strains of *Staphylococcus* species [23]. Due to reports of nosocomial infections with these pathogens, they are of serious public health concern as they may contaminate the hospital environment and can spread to other animal patients [24]. Considering the ESBL-producing *E. coli,* it is important to limit the genetic pressure toward antimicrobial resistance development by implementing microbiological monitoring of all cutaneous infections and rational use of antimicrobials [25].

A large number of veterinarians used quinolones to treat urinary tract infections followed by cephalosporins of the 3rd and 4th generation. The use of quinolones should be limited only to upper urinary tract infection (pyelonephritis) in dogs and cats and the cases of cystitis should be treated using amoxicillin or trimethoprim/sulphonamides, after proper microbiological confirmation of bacteria and AST [13]. Studies in India have reported frequent isolation of ESBL-producing members of *Enterobacteriaceae* from companion animals, which is of serious concern due to the risk of transmission to owners [7].

For respiratory infections, tetracyclines are usually recommended as first choice; however, findings of the current study are in conflict, as quinolones were found to be the preferred choice. Similarly, in place of penicillin, cephalosporins of the 3rd and 4th generations were the second choices [13]. The use of cephalosporins of the 3rd and 4th generations for such infections is of great concern as these antimicrobials are of critical importance for human medicine [26]. In reproductive infections, cephalosporins of the 3rd and 4th generations were preferred, followed by quinolones and penicillin, and this does not comply with the guidelines, except for penicillin. For GIT infections, sulphonamides were preferred and cephalosporins of the 3rd and 4th generations were the second choices of the respondents, which is not in consensus with the guidelines. For sepsis, penicillin was the first choice of the respondents, which is in line with the guidelines. For eye-related ailments veterinarians preferred aminoglycosides and quinolones (fluoro) over the guideline recommendations of tetracycline or chloramphenicol. Usually, the prescription of systemic antimicrobials for eye- and ear-related ailments is not recommended until any systemic pathogen is involved in the pathogenesis. The respondents in the current study might have reported the use of topical antimicrobials for eye and ear ailments, leading to deviation from the guidelines. The excessive use of topical antimicrobials also serves as a key driver for emergence of AMR in *S*. *aureus* isolates [27]. The guidelines might vary based on the resistance patterns observed at various geographical locations; however, the findings of the current study warrant the necessity of formulation and strict implementation of guidelines on antimicrobial usage in India. The use of antimicrobials listed as HPCIA by the WHO is a major concern and significantly higher prescription of such drugs by practitioners having less clinical experience might be attributed to marketing by some of the pharmaceutical companies or practitioners’ own desire for faster results or recovery [28,29].

The duration of treatment for various organ systems lies in line with the proposed guidelines as reported by most of the practitioners. For respiratory diseases, 71% of the veterinarians stated a usual treatment duration of 3–7 days, in line with the recommended duration of 5–7 days [13], and the same duration was observed for urinary tract infections, as reported by more than half of the veterinarians (54%). For GIT infections, three fourths of the practitioners (75%) used antimicrobials for 3–7 days, consistent with the guidelines [13,30]. For infections related to skin, 60% of respondents recommended at least 14 days or more, near to the recommended guidelines, and around 37% reported a treatment duration of 7 days. For ear infections, a lesser number of respondents (30%) reported the recommended practice (up to 14 days) guidelines. For eye infections, more than half of the veterinarians (56%) reported a duration of 3–7 days, which is far less than the recommended guidelines of 4–5 weeks [13]. For other organ systems (reproductive and sepsis), the treatment duration was similar to the recommended guidelines.

Macrolides and cephalosporins of the 3rd and 4th generations and are listed as critically important antimicrobials of the highest priority, whereas, aminoglycosides and carbapenems are listed as antimicrobials of high priority. The use of these classes of antibiotics has been limited to non-responsive human cases and, as per the recommendations, the use of such antibiotics should be done only after AST [26]. The frequent use of such antimicrobials indicates a serious lack of the best practice protocols, as observed in the current study, and warrants an urgent need to formulate antimicrobial usage guidelines by veterinary authorities.

It is a well-accepted concept that there is no requirement of antimicrobials in patients undergoing a clean or clean-contaminated procedure who are at low risk (healthy or with localized disease) and/or apyretic with systemic illness [31]. Around three fourths of veterinarians reported the use of antimicrobials in clean surgical procedures and that too in more than 50% of the cases presented before them, just to prevent potential post-surgical infections. Considering the available literature and guidelines, it can be inferred that most of the respondents seems to be unaware of best practices in this aspect. A national action plan to contain the AMR in India was formulated for implementation in the year 2017, but the efforts seem insufficient as it does not formally address the use of antimicrobials in veterinary medicine, and especially in small animal practice.

The present study also has some limitations in terms of a low response rate, region covered, and lack of subjective questions seeking practitioner’s aspects on antimicrobial practices and solutions. The survey is the first of its kind to have a comprehensive approach regarding attitude, opinions, and working preferences among pet practitioners relating to antimicrobials. Moreover, the survey also provides future insight into how to conduct such studies to address the menace of antimicrobial resistance among veterinarians, pet owners, dairy farmers, and the general public.

## 4. Materials and Methods

### 4.1. Study and Questionnaire Design

A cross-sectional questionnaire survey with 33 parameters was designed to collect the information on antimicrobial prescription, duration of treatment with antimicrobials, and organ or disease-specific antimicrobial prescription practices among pet practitioners in India. A comprehensive review of the literature was conducted to identify the antimicrobial prescription patterns [10,32] and various factors responsible for its irrational use in pet practice [7,13,33]. The survey instrument was designed by the research team at the department. To ensure maximum participation, simple questions were used, and it was designed to take approximately 15–20 min for completion. The questionnaire was divided into four sections and all the sections contained closed-ended questions with a combination of Yes/No questions, multiple-choice questions, and questions related to the frequency of events, except the first section, which sought demographic information. The second section of the questionnaire (17 questions) included questions seeking information on various aspects of antimicrobial prescription and the pet owner’s compliances, whereas the third and fourth sections included questions (08 question each) on use of antimicrobials as per the disease or organ system and duration of the treatment regimen, respectively. Initially, an evaluation of the questionnaire was conducted to identify all the important issues to be addressed and also to remove potentially ambiguous questions. Formal testing of the questionnaire on a small group of respondents (*n* = 10) from the intended study population was carried to remove confusing, ambiguous, or misleading questions and simultaneously inclusion of additional response categories for multiple-choice questions.

The efforts were made to cover all the aspects of antimicrobial usage and to keep the questionnaire consistent with similar studies from other parts of the globe, to ensure comparison of the results [14,15,17]. Most of the questions aimed to identify the frequency of the events of interest; e.g., the proportion of antimicrobial treatment non-compliance by pet owners (never 0%, sometimes 30%, frequently 70%, always 100%). To understand the use of antimicrobials to treat ailments involving specific organ systems, the questionnaire was designed to know the information on the class of antimicrobials or the active compound of preference.

### 4.2. Sampling Procedure

The source population for the present study comprised registered veterinarians (Veterinary Council of India and/or State Veterinary Council) of India, and the study population included veterinarians who fulfilled the inclusion criteria of being pet practitioners. The sample size was calculated using the ‘Raosoft calculator’ (http://www.raosoft.com/samplesize.html?nosurvey, accessed on 27 August 2021). The sample size of 91 was estimated based on a 60% response distribution [32], a 10% margin of error, and a 95% confidence interval. The expected response proportion of 60% was assumed based on the results of the study by [32] on large animal practitioners in India. So, a total of 200 questionnaires were sent to the veterinarians selected through registered emails and/or personal contacts from professional societies and social media groups. Only one practitioner per clinic/hospital was included in the study. The questionnaire was circulated using the online interface of Google Forms (Google LLC, Mountain View, CA, USA) to the target population, and the survey remained open for a period of four months from September 2021 to December 2021.

### 4.3. Assessing Compliance with the Guidelines

The guidelines issued by the WHO and DSAVA on antibiotic use were considered to assess the compliance of practitioners as no official guidelines on prescriptions of antimicrobials in veterinary practice were available in the country for comparison.

### 4.4. Statistical Analysis

The completed questionnaires were manually checked for data quality and code was assigned to responses in Microsoft^®^ Office Excel 2010 for statistical analysis. Chi-square tests were used to observe the association between the experience of veterinarians in years (less than 5 years; 5–15 years and more than 15 years) and the written antimicrobial policy at clinics, weighing the animals before antimicrobial prescription, method of selecting antimicrobials, dose rate application, human antimicrobial prescription pattern, and antimicrobials use after clean surgical operations, etc.

## 5. Conclusions

From the results of the survey, a number of recommendations for the rationalization of antimicrobial use in small animal practice in India can be made. At first, practitioners need subject guidance on this aspect. There is an urgent need for official guidelines on antimicrobials in small animal practice by national/state-level authorities. The guidelines should be specified as per organ system and duration of treatment. Secondly, and more urgently, a systematic AMR surveillance mechanism need to be established for dispersing the information related to initial choice of antimicrobials based on local resistance profiles. Third, there is a need to educate pet owners about antimicrobial resistance and the importance of rational use of antimicrobials for their pets. Over-the-counter availability of antimicrobials without prescription need to be restricted. Fourth, the National Action Plan on Antimicrobial Resistance prepared by the Ministry of Health and Family Welfare, Government of India, and others, such as The Chennai Declaration to tackle AMR, must include more veterinarians as members to address the issue suitably. In the absence of discussions between various stakeholders, animal husbandry activities are unnecessarily blamed for the emergence and dispersion of antimicrobial resistance. Finally, the ‘One Health’ approach, facilitating multi-stakeholder collaborations to formulate novel strategies/interventions for promoting antimicrobial stewardship through AMR education campaigns, is the need of the hour.

## Figures and Tables

**Table 1:** Descriptive statistics of the responses provided by the respondents (*n* = 104).

Items	Options	Respondents	
		Frequency	Percentage
Gender	Male	74	71.2
	Female	30	28.8
Age (in years)	24–34	80	76.9
	35–50	19	18.3
	>50	5	4.8
Type of clinic	Private	56	53.8
	University/college clinics	20	19.2
	Government Veterinary Hospital (GVH)	28	26.9
Years of experience	0–5	71	68.2
	5–15	18	17.3
	>15	15	14.4
Number of vets	1	51	49.0
	2–5	31	29.8
	>5	22	21.2
Written policy on antimicrobials	Yes	46	44.2
	No	58	55.8
Facility for antimicrobial sensitivity testing at clinics	Yes	37	35.6
	No	67	64.4
Dose rate calculation of antimicrobial to be used as per	The condition	64	61.5
	General principle	40	38.5
Owner-initiated treatments before a case is presented at clinic/hospital	Never (0%)	5	4.8
	Sometimes (0–30%)	68	65.4
	Frequently (70%)	31	29.8
How often combinations of different antimicrobials are prescribed?	Infrequently (very rarely)	70	67.3
	Frequently	30	28.8
	Never	4	3.8
Encounter of owner compliance challenges	Frequently (70%)	39	37.5
	Always (100%)	6	5.8
	Sometimes (30%)	58	55.8
	Never	1	0.9
Preferred method of antimicrobial selection in relevance to antimicrobial sensitivity results	Empirical, whilst awaiting antibiogram	26	25.0
	Empirical first, antibiogram if unsuccessful	54	51.9
	Antibiogram first	5	4.8
	I rarely use antibiogram	19	18.3
Basis of antimicrobial selection	Bibliography (e.g., veterinary index/subject books)	41	39.4
	Leaflet indications	3	2.9
	Own professional experience	44	42.3
	Antimicrobial sensitivity testing	10	9.6
	Discussion with colleagues	6	5.8
Prescription of antimicrobial while waiting for laboratory results	Never (0%)	7	6.7
	Sometimes (0–3%)	55	52.9
	Frequently (30–70%)	33	31.7
	Always (100%)	9	8.7
Use of antimicrobials exclusively meant for humans.	Never (0%)	32	30.8
	Sometimes (0–30%)	54	51.9
	Frequently (30–70%)	13	12.5
	Always (100%)	5	4.8
Maintenance of client’s visit and prescription record with respect to antimicrobial prescribed?	Yes	91	87.5
	No	13	12.5
If answer to the question above is no, then please state the reason	Lack of Time	3	23.1
	Not Important	2	15.4
	Both of above	8	61.5
Practice of weighing the animal before prescribing antimicrobials	Always	61	58.7
	Frequently	18	17.3
	Sometimes	19	18.3
	Never	6	5.8
How often sick animals need to bring to clinics again because of antimicrobial treatment failure?	Never	10	9.6
	Sometimes (1–3 times/year)	79	76.0
	Frequently (>3 times/year)	15	14.4
How often postoperative antimicrobials are prescribed after clean surgical operations?	0% of cases	3	2.9
	1–10% of cases	13	12.5
	11–50% of cases	11	10.6
	51–90% of cases	21	20.2
	>90% of cases	56	53.8
If answer to the previous reason is more than 50%, then may specify the reasons for prescribing postoperative antimicrobials?	Just the typical procedure	20	25.9
	Operations last more than 90 min	6	7.8
	Frequent issues with aseptic procedures	18	23.4
	Frequent postoperative infections	31	40.2
	Other Reasons	2	2.6

**Table 4:** Correlation between pet practitioner’s years of experience and other variables.

Items	Options	Years of Experience			*p* Value (Chi-Square Test)
		0–5 Years (*n* = 71)	5–15 Years (*n* = 18)	>15 Years (*n* = 15)	
Written antimicrobial usage policy	Yes	32 (45.1)	6 (33.3)	8 (53.3)	0.475
	No	39 (54.9)	12 (66.7)	7 (46.7)	
Weighing the animal before prescribing antimicrobials	Always (100%)	41 (57.8)	10 (55.6)	10 (66.7)	0.730
	Often (50%)	26 (36.6)	6 (33.3)	5 (33.3)	
	Never (0%)	4 (5.6)	2 (11.1)	0	
Method of selecting antimicrobials	Empirical, whilst awaiting antibiogram	18 (25.4)	6 (33.3)	2 (13.3)	0.619
	Empirical first, antibiogram if unsuccessful	35 (49.3)	9 (50.0)	10 (66.7)	
	Antibiogram first	5 (7.0)	0	0	
	Antibiogram rarely used	13 (18.3)	3 (16.7)	3 (20.0)	
How often postoperative antimicrobials are prescribed after clean surgical operations?	0% of cases	3 (4.2)	0	0	0.136
	1–10% of cases	7 (9.9)	3 (16.7)	3 (20.0)	
	11–50% of cases	7 (9.9)	3 (16.7)	1 (6.6)	
	51–90% of cases	12 (16.9)	2 (11.1)	7 (46.7)	
	>90% of cases	42 (59.1)	10 (55.5)	4 (26.7)	
Follow dose rate of antimicrobials as per	The condition	42 (59.1)	11 (61.1)	11 (73.3)	0.650
	General principle	29 (40.9)	7 (38.9)	4 (26.7)	
Facility for an tibiogram	Yes	29 (40.9)	5 (27.8)	3 (20.0)	0.232
	No	42 (59.1)	13 (72)	12 (80.0)	
How often do you prescribe the combination of different antimicrobials?	Infrequently	48 (67.6)	11 (61.1)	11 (73.3)	0.591
	Frequently	19 (26.8)	7 (38.9)	4 (26.7)	
	Never	4 (5.6)	0	0	
How often do you encounter the owner compliance challenges	Sometimes (30%)	37 (52.1)	11 (61.1)	10 (66.7)	0.688
	Frequently (70%)	27 (38.0)	7 (38.9)	5 (33.3)	
	Always (100%)	6 (8.5)	0	0	
	Never	1 (1.4)	0	0	
Choice of the antibiotic is based mainly on	Bibliography (e.g., veterinary index/subject books)	29 (40.9)	7 (38.9)	5 (33.3)	0.696
	Own professional experience	27 (38.0)	8 (44.4)	9 (60.0)	
	Antimicrobial sensitivity testing	7 (9.9)	2 (11.1)	1 (6.7)	
	Discussion with colleagues/leaflet	8 (11.2)	1 (5.6)	0	
How often you use antibiotics that are exclusively meant for humans?	Never (0%)	25 (35.2)	7 (38.9)	1 (6.7)	0.020 *
	Sometimes (0–30%)	39 (54.9)	9 (50.0)	8 (53.3)	
	Frequently (30–70%)	7 (9.9)	2 (11.1)	5 (33.3)	
	Always (100%)	0	0	1 (6.7)	

The values in parentheses are percentages, unless stated otherwise. * Significant association (*p* < 0.05).

## Data Availability

All the supporting information has been provided in the text itself. Any other supporting files if requested can be provided by the corresponding author.
